# Mode of presentation and mortality amongst patients hospitalized with heart failure? A report from the First Euro Heart Failure Survey

**DOI:** 10.1007/s00392-018-1380-6

**Published:** 2018-10-25

**Authors:** Ahmad Shoaib, M. Farag, J. Nolan, A. Rigby, A. Patwala, M. Rashid, C. S. Kwok, R. Perveen, A. L. Clark, M. Komajda, J. G. F. Cleland

**Affiliations:** 1 Keele Cardiovascular Research Group, Institute of Applied Clinical Sciences and Centre for Prognosis Research, Keele University, Stoke on Trent, UK; 2 Department of Academic Cardiology, University of Hull, Kingston upon Hull, UK; 3 Postgraduate Medical School, University of Hertfordshire, Hertfordshire, UK; 4 Department of Cardiology, University of Pierre and Marie Curie Paris VI, La Pitié-Salpêtrière Hospital, Paris, France; 5 Robertson Centre for Biostatistics, University of Glasgow and National Heart and Lung Institute, Imperial College London, London, UK

**Keywords:** Acute heart failure, Presentation of heart failure, Mortality

## Abstract

**Background:**

Heart failure is heterogeneous in aetiology, pathophysiology, and presentation. Despite this diversity, clinical trials of patients hospitalized for HF deal with this problem as a single entity, which may be one reason for repeated failures.

**Methods:**

The first EuroHeart Failure Survey screened consecutive deaths and discharges of patients with suspected heart failure during 2000–2001. Patients were sorted into seven mutually exclusive hierarchical presentations: (1) with cardiac arrest/ventricular arrhythmia; (2) with acute coronary syndrome; (3) with rapid atrial fibrillation; (4) with acute breathlessness; (5) with other symptoms/signs such as peripheral oedema; (6) with stable symptoms; and (7) others in whom the contribution of HF to admission was not clear.

**Results:**

The 10,701 patients enrolled were classified into the above seven presentations as follows: 260 (2%), 560 (5%), 799 (8%), 2479 (24%), 1040 (10%), 703 (7%), and 4691 (45%) for which index-admission mortality was 26%, 20%, 10%, 8%, 6%, 6%, and 4%, respectively. Compared to those in group 7, the hazard ratios for death during the index admission were 4.9 (*p* ≤ 0.001), 4.0 (*p* < 0.001), 2.2 (*p* < 0.001), 2.1 (*p* < 0.001), 1.4 (*p* < 0.04) and 1.4 (*p* = 0.04), respectively. These differences were no longer statistically significant by 12 weeks.

**Conclusion:**

There is great diversity in the presentation of heart failure that is associated with very different short-term outcomes. Only a minority of hospitalizations associated with suspected heart failure are associated with acute breathlessness. This should be taken into account in the design of future clinical trials.

## Introduction

Acute heart failure (AHF) is heterogeneous in its aetiology, pathophysiology, and presentation. European Society of Cardiology (ESC) 2008 guidelines classify AHF into six different clinical presentations, amongst which there is considerable overlap. Most events are classified as decompensated chronic heart failure and the rest as acute pulmonary oedema, cardiogenic shock, right heart failure or associated with severe hypertension or acute coronary syndrome [1]. It is clear that AHF is not a discrete diagnosis but a collection of different clinical syndromes that require clinical intervention with varying degrees of urgency [2, 3]. A patient with severe breathlessness at rest in acute pulmonary oedema is a medical emergency requiring immediate investigation and treatment [4, 5]. A patient presenting with increasing exertional breathlessness and worsening peripheral oedema might be considered sub-acute and requiring treatment within hours or days rather than minutes [6, 7]. If the target and purpose of therapy is diverse then trials that treat all AHF as a single entity are likely to fail. Better characterization of the heterogeneous clinical presentation of AHF might help inform the design of future clinical trials that target the unmet needs of specific presentations of AHF [8–10]. Accordingly, we obtained information from the First EuroHeart Failure Survey (EHFS-1) that enrolled more than 10,000 patients from 115 hospitals over a 6 week snapshot to describe the outcome of patients with different presentations of patients hospitalized for or with heart failure [11, 12].

## Methods

EHFS-1 screened consecutive deaths and discharges during 2000–2001 primarily from medical wards over a 6 week period in 115 hospitals from 24 countries in Europe, to identify patients with known or suspected heat failure (HF). The design and implementation of the survey have been published in detail previously [13]. Each hospital recorded consecutive deaths and discharges from medical, cardiology, cardiac surgery, and geriatric medicine wards over a period of 6 weeks [11]. Surgical, gynaecology, ophthalmology, and renal wards were excluded. Records were screened to identify if the patient fulfilled one or more of the following four criteria:

1. A clinical diagnosis of HF, primary or contributory, during the index admission.
2. A diagnosis of HF recorded in the hospital records at any time during the previous 3 years.
3. Administration of loop diuretics during the 24 h prior to death or discharge during the index admission, other than for end-stage renal disease.
4. Administration of treatment for HF or for ventricular dysfunction within the 24 h prior to death or discharge.

Patients who fulfilled one or more inclusion criteria were further classified by investigators according to clinical presentation, aetiology, final diagnosis and whether, in the investigators opinion, HF was the primary diagnosis, a secondary diagnosis complicating or prolonging hospital admission, an incidental finding (e.g. patients who were admitted for another reason and whose admission was not complicated or prolonged by HF) or diagnostically uncertain (mostly patients taking loop diuretics for no obvious reason).

### Classification of presentation

Presentation at hospital admission was classified hierarchically (patients belonging to a preceding class/group could not belong to any subsequent class/group) as follows:

1. Cardiac arrest, ventricular tachycardia, or fibrillation or cardiogenic shock.
2. Acute coronary syndrome (ACS).
3. Atrial fibrillation (AF) with a rapid ventricular response (> 120/min).
4. Severe shortness of breath at rest.
5. Other symptoms of HF, such as worsening peripheral oedema.
6. Stable symptoms.
7. Contribution of HF to admission uncertain.

Detailed information regarding events contributing to the current admission, cardiovascular and non-cardiovascular comorbid illnesses, and clinical investigations during admission and therapy at discharge or 24 h prior to death were recorded by investigators as well as deaths during the index hospital admission and deaths and readmissions within 12 weeks after discharge.

For most patients, left ventricular systolic dysfunction (LVSD) was not measured formally but assessed in a semi-quantitative fashion. For guidance, a left ventricular ejection fraction (LVEF) of < 40% was considered to reflect moderate-to-severe LVSD.

Continuous data are summarized by the median and 25th/75th percentiles; categorical data by percentages. As time-to-event data were not recorded after discharge, prognostic models for all-cause mortality were developed using Logistic regression. Prognostic models were developed using *k*-fold cross validation [14]. This procedure splits the data randomly into *k* partitions. For each partition, it fits the specified model using the other *k* − 1 groups, and uses the resulting parameters to predict the dependent variable in the unused group [15, 16]. We arbitrarily choose k as 25 (hence 25-fold cross validation). We started with 50 clinically relevant variables and then selected those variables in the final model that remained significant for at least 70% of cross validations. The significance level to remain in the model was initially set to 0.05 for each model. From the logistic regression models, receiver operating characteristic (ROC) curves were plotted as sensitivity versus 1 − specificity. An area under the ROC curve was calculated using methods outlined in Hanley and McMeil [17]. The area under the ROC represents the probability of classifying an individual as dead/alive. An area under the ROC curve of 1.0 means perfect classification, while an area of 0.5 means that classification is no better than chance. The Stata 13 statistical computer package was used to analyse the data.

## Results

HF was the primary diagnosis in 4234 (40%) patients, a secondary diagnosis in 1772 (17%), and was considered not to have caused or complicated the index admission in 4695 (44%) [18]. Of the 6006 patients in whom HF was thought to cause or complicate admission, the most common presentation was severe breathlessness at rest (*n* = 2479; 42% of such patients) (Table 1).

**Table 1:** Clinical characteristics

	Group 1	Group 2	Group 3	Group 4	Group 5	Group 6	Group 7
Presentation	Arrest/VT/Shock	ACS	Rapid AF	ASOB	Other/oedema	Stable	Uncertain
Numbers (%)	260 (2%)	560 (5%)	799 (8%)	2479 (24%)	1040 (10%)	703 (7%)	4695 (44%)
Age in years (IQR)	69 (61–76)	73 (66–81)	75 (66–82)	74 (66–80)	72 (62–79)	66 (56–76)	73 (64–80)
Women (%)	90 (35%)	240 (43%)	424 (53%)	1185 (48%)	457 (44%)	258 (37%)	2293 (49%)
BMI (kg/m^2^) (IQR)	26 (24–29)	26 (24–29)	26 (23–30)	26 (23–29)	26 (24–30)	26 (24–30)	27 (24–30)
Prior HF admission (%)	89 (38%)	92 (35%)	322 (40%)	1136 (46%)	429 (41%)	320 (46%)	708 (15%)
Loop diuretics prior to admission (%)	131 (59%)	184 (36%)	408 (55%)	1651 (71%)	665 (70%)	530 (79%)	2395 (58%)
Loop diuretics prior to death or discharge (%)	211 (82%)	428 (77%)	667 (84%)	2177 (88%)	748 (72%)	539 (77%)	3455 (74%)
ACS—this admission	87 (34%)	493 (89%)	13 (2%)	43 (2%)	9 (1%)	10 (1%)	413 (9%)
MI (anytime)	146 (56%)	530 (95%)	183 (23%)	782 (32%)	335 (32%)	213 (31%)	1746 (37%)
Any IHD (ACS/Revasc)							
DCM	48 (19%)	32 (6%)	88 (11%)	380 (15%)	128 (12%)	181 (26%)	336 (7%)
Valve replacement/repair	12 (5%)	14 (3%)	62 (8%)	130 (5%)	67 (6%)	59 (8%)	290 (6%)
AF (%)	111 (43%)	188 (34%)	765 (96%)	1006 (41%)	415 (40%)	239 (34%)	1738 (37%)
Prior H/O VT/VF	148 (58%)	77 (14%)	62 (8%)	134 (5%)	72 (7%)	74 (11%)	296 (6%)
Pacemaker	41 (16%)	31 (6%)	51 (6%)	219 (9%)	96 (9%)	81 (12%)	347 (7%)
ICD	29 (11%)	4 (1%)	1 (0.1%)	22 (1%)	17 (2%)	17 (2%)	60 (1%)
H/O hypertension	132 (52%)	329 (60%)	398 (50%)	1377 (56%)	550 (53%)	347 (50%)	2452 (53%)
Disabling stroke	17 (7%)	53 (10%)	45 (6%)	199 (8%)	111 (11%)	64 (9%)	438 (9%)
Minor stroke/TIA	26 (10%)	50 (9%)	70 (9%)	230 (9%)	87 (8%)	65 (9%)	539 (12%)
H/O renal dysf	72 (28%)	124 (22%)	145 (18%)	554 (22%)	193 (19%)	131 (19%)	593 (13%)
H/O resp. disease	63 (25%)	147 (27%)	289 (36%)	985 (40%)	297 (29%)	166 (24%)	1392 (30%)
Diabetes mellitus	56 (22%)	147 (26%)	171 (22%)	749 (30%)	279 (27%)	197 (28%)	1178 (25%)
H/O PE	11 (4%)	15 (3%)	29 (4%)	92 (4%)	37 (4%)	17 (2%)	145 (3%)
Clinical investigations							
Echo data available	177	376	513	1562	591	498	2339
Moderate/severe LVSD (ejection fraction < 40%)	122 (69%)	240 (64%)	245 (48%)	840 (54%)	346 (59%)	317 (64%)	951 (41%)
Moderate/severe LV dilatation	72 (41%)	78 (21%)	129 (25%)	514 (33%)	236 (40%)	202 (41%)	462 (20%)
Moderate/severe LA dilatation	59 (33%)	82 (22%)	194 (38%)	605 (39%)	247 (42%)	195 (39%)	574 (25%)
Moderate/severe mitral regurgitation	53 (30%)	100 (27%)	192 (37%)	559 (36%)	247 (42%)	180 (36%)	574 (25%)
Moderate/severe aortic stenosis	7 (4%)	20 (5%)	39 (8%)	157 (10%)	62 (10%)	43 (9%)	163 (7%)
Haemoglobin (g/dl) (IQR)	12.5 (11–14.2)	12.7 (11.2–13.9)	12.7 (1.1–14.2)	12.7 (11.3–14)	13.3 (11.8–14.5)	13.1 (1.5–14.7)	12.9 (11.3–14.2)
Sodium (mmol/l) (IQR)	139 (135–142)	139 (136–142)	139 (136–142)	139 (136–142)	140 (137–142)	139 (136–142)	139 (136–142)
Potassium (mmol/l) (IQR)	4.2 (3.8–4.6)	4.2 (3.8–4.6)	4.2 (3.9–4.6)	4.2 (3.9–4.6)	4.4 (4–4.8)	4.3 (4–4.7)	4.2 (3.9–4.6)
Urea mmol/l (IQR)	12.9 (6.9–20.7)	10.7 (6.8–17)	10.4 (7.1–17.5)	11.8 (7.5–18.6)	9.4 (6.6–15)	10.5 (6.9–17.1)	8.9 (6.2–14.5)
Creatinine (μmol/l) (IQR)	124 (99–168)	106 (88–137)	106 (85–133)	106 (88–138)	106 (88–134)	106 (88–135)	101 (83–126)
eGFR 30–60 ml/min							
eGFR < 30 ml/min							
Cholesterol most recent (mmol/l) (IQR)	5.1 (4–5.8)	5.1 (4.3–6)	4.7 (3.8–5.6)	4.9 (4–5.8)	4.9 (3.9–5.9)	5.1 (4.1–5.9)	5.1 (4.3–5.9)
Chest X-ray: cardiomegaly/pulmonary congestion	205 (94%)	392 (79%)	618 (86%)	1938 (88%)	717 (79%)	457 (76%)	2281 (61%)

*HF* heart failure, *ACS* acute coronary syndrome, *AF* atrial fibrillation, *ASOB* acute shortness of breath, *Asymp. LVD* asymptomatic left ventricle dysfunction, *BMI* body mass index, *MI* myocardial infarction, *USA* unstable angina, *PCI* percutaneous coronary intervention, *CABG* coronary artery bypass grafting, *DCM* dilated cardiomyopathy, *SVT* supraventricular tachycardia, *VT* ventricle tachycardia, *VF* ventricle fibrillation, *TIA* transient ischaemic attack, *DM* diabetes mellitus, *PE* pulmonary embolism, *IQR* interquartile range, *LVEDD* left ventricle end diastolic diameter, *LVESD* left ventricle end systolic diameter, *LV* left ventricle, *LA* left atrium

The age and sex distribution was broadly similar for the various patient presentations, but patients presenting with a cardiac arrest or in shock and those with stable symptoms were slightly younger and were more likely to be men (Table 1). Apart from those admitted with a cardiac arrest/shock or ACS, about one-third of patients had a prior history of myocardial infarction. AF was present in 34–43% of patients, not including those patients with rapid AF as their primary presentation. For each group, apart from ACS, > 50% had been treated with loop diuretics prior to admission and prescription at discharge ranged from 72 to 88% amongst groups. When echocardiographic information was available (6096 patients), moderate-to-severe left ventricular systolic dysfunction (LVSD) was reported in 41–69% of cases and moderate-to-severe mitral regurgitation in 25–42% (Table 1). Even when the contribution of HF to admission was uncertain, 41% were reported to have moderate-to-severe LVSD, 25% moderate-to-severe mitral regurgitation, 61% cardiomegaly or pulmonary congestion on a chest X-ray, 74% were prescribed loop diuretics, 60% an ACE inhibitor or angiotensin receptor blocker, and 38% a beta-blocker (Table 2), making a diagnosis of heart failure likely in many cases.

**Table 2:** Drugs at discharge or 24 h prior to death and mortality

	Group 1	Group 2	Group 3	Group 4	Group 5	Group 6	Group 7
Presentation	Arrest/VT/shock	ACS	Rapid AF	ASOB	Other/oedema	Stable	Uncertain
Numbers (%)^a^	260 (2%)	560 (5%)	799 (8%)	2479 (24%)	1040 (10%)	703 (7%)	4695 (44%)
Spironolactone	61 (23%)	94 (17%)	203 (25%)	693 (28%)	351 (34%)	230 (33%)	540 (12%)
Furosemide	202 (78%)	436 (78%)	663 (83%)	2121 (86%)	774 (74%)	511 (73%)	3327 (71%)
Bumetanide	7 (3%)	7 (1%)	26 (3%)	97 (4%)	19 (2%)	12 (2%)	110 (2%)
Torasemide	14 (5%)	19 (3%)	38 (5%)	93 (4%)	30 (3%)	31 (4%)	140 (3%)
Metolazone	0	2 (0.4%)	11 (1%)	61 (2%)	16 (2%)	4 (1%)	21 (0.5%)
Thiazide diuretic	33 (13%)	41 (7%)	69 (9%)	219 (9%)	198 (19%)	96 (14%)	397 (8%)
ACEI	158 (61%)	399 (71%)	494 (62%)	1655 (67%)	735 (71%)	496 (71%)	2577 (55%)
ARB	7 (3%)	18 (3%)	28 (4%)	114 (5%)	47 (5%)	51 (7%)	213 (5%)
Nitrate	106 (41%)	316 (56%)	269 (34%)	1137 (46%)	525 (50%)	258 (37%)	2005 (43%)
CCB	30 (12%)	119 (21%)	178 (22%)	483 (19%)	184 (18%)	107 (15%)	1131 (24%)
Beta blockers	115 (44%)	309 (55%)	256 (32%)	676 (27%)	433 (42%)	288 (41%)	1790 (38%)
Digoxin	87 (33%)	140 (25%)	501 (63%)	1059 (43%)	469 (45%)	296 (42%)	1227 (26%)
Antiarrhythmic drugs	85 (33%)	96 (17%)	244 (31%)	300 (12%)	104 (10%)	127 (18%)	599 (13%)
Lipid lowering drugs	38 (15%)	147 (26%)	83 (10%)	420 (17%)	192 (18%)	159 (23%)	1097 (23%)
Mortality and length of stay during index admission							
Deaths	67 (26%)	114 (20%)	80 (10%)	201 (8%)	65 (6%)	41 (6%)	189 (4%)
HR compared to class 7 (uni-variable analysis)	4.86 (*p* ≤ 0.001, CI 3.57–6.6)	3.95 (*p* ≤ 0.001, CI 3.1–5)	2.22 (*p* ≤ 0.001, CI 1.7–2.9)	2.09 (*p* ≤ 0.001, CI 1.70–2.56)	1.36 (*p* = 0.04, CI 1.02–1.81)	1.44 (*p* = 0.04, CI 1.02–2.02)	
Events contributing to death (proportion deaths)							
MI (%)	32 (47%)	98 (86%)	5 (6%)	11 (5%)	4 (6%)	3 (7%)	31 (16%)
Worsening HF	45 (67%)	81 (71%)	60 (75%)	167 (83%)	52 (80%)	21 (51%)	35 (18%)
Renal dysfunction	20 (30%)	33 (29%)	18 (23%)	54 (27%)	19 (29%)	10 (24%)	21 (11%)
Ventricular arrhythmia	18 (27%)	21 (18%)	14 (18%)	18 (9%)	3 (5%)	3 (7%)	13 (7%)
Atrial arrhythmia	5 (7%)	15 (13%)	30 (38%)	14 (7%)	5 (8%)	4 (10%)	8 (4%)
Infection	13 (5%)	18 (16%)	26 (33%)	65 (32%)	31 (48%)	14 (34%)	57 (30%)
Stroke	3 (5%)	5 (4%)	2 (3%)	10 (5%)	10 (15%)	6 (15%)	32 (1%)
Cancer	0	2 (2%)	5 (6%)	16 (8%)	2 (3%)	2 (5%)	30 (16%)
Other	11 (16%)	13 (11%)	22 (28%)	45 (22%)	12 (18%)	10 (24%)	72 (38%)
LoS-index admission (days) (median/IQR)	9 (4–16)	11 (7–18)	10 (6–15)	8 (5–13)	10 (5–17)	8 (3–14)	8 (4–13)
Mortality and readmission within 12 weeks after discharge							
Number at risk^∗^	193	446	719	2278	975	662	4506
Deaths after discharge	12 (6%)	36 (8%)	47 (7%)	175 (8%)	63 (6%)	41 (6%)	229 (5%)
Unadjusted OR compared to class 7	1.18 (*p* = 0.58, CI 0.64–2.15)	1.56 (*p* = 0.02, 1.08–2.25)	1.26 (*p* = 0.17, CI 0.91–1.75)	1.46 (*p* ≤ 0.001, CI 1.19–1.79)	1.14 (*p* = 0.37, CI 0.86–1.53)	1.18 (*p* = 0.35, CI 0.84–1.68)	
Events contributing to death (proportion deaths)							
MI (%)	1 (8%)	10 (28%)	4 (9%)	12 (7%)	5 (8%)	2 (5%)	25 (11%)
Worsening HF (%)	3 (25%)	9 (26%)	10 (21%)	68 (39%)	25 (40%)	17 (41%)	50 (22%)
Renal dysf. (%)	0	2 (6%)	3 (6%)	19 (11%)	3 (5%)	6 (15%)	12 (6%)
Arrhythmia (%)	1 (8%)	3 (9%)	2 (4%)	7 (4%)	3 (5%)	3 (7%)	7 (3%)
Infection (%)	0	3 (9%)	6 (13%)	14 (8%)	8 (12%)	4 (10%)	40 (17%)
Stroke	0	1 (3%)	8 (17%)	10 (6%)	4 (6%)	3 (7%)	19 (8%)
Cancer	0	1 (3%)	3 (6%)	11 (6%)	6 (9%)	3 (7%)	28 (12%)
Other	1 (8%)	7 (23%)	16 (34%)	32 (18%)	7 (11%)	5 (12%)	63 (28%)
Readmission within 12 weeks after discharge							
Number at risk^∗^	193	446	719	2278	975	662	4506
All cause	43 (22%)	109 (24%)	166 (23%)	557 (24%)	189 (19%)	192 (29%)	980 (22%)
Due to CV cause	33 (17%)	85 (19%)	131 (18%)	421 (18%)	155 (16%)	150 (23%)	580 (13%)
Due to heart failure	20 (10%)	45 (10%)	73 (10%)	298 (13%)	98 (10%)	98 (15%)	240 (5%)

*HF* heart failure, *ACS* acute coronary syndrome, *AF* atrial fibrillation, *ASOB* acute shortness of breath, *ACI* angiotensin converting enzyme inhibitor, *ARB* angiotensin receptor blockers, *LOS* length of stay, *MI* myocardial infarction, *IQR* interquartile range, *CV* cardiovascular

^a^Data from 9779 patients were available for the 12-week follow-up period

Mortality during the index admission was higher for those presenting with a ventricular arrhythmia, cardiac arrest or shock (26%), or an ACS (20%) (Table 2). The group for which HF made an uncertain contribution to admission had a lower in-patient mortality (4%). Median length of stay varied from 8 to 11 days amongst groups. Of 10,701 patients, 12 weeks follow-up data were available for 9779 (91%). Mortality in the 12 weeks after discharge varied little amongst groups, ranging from 5 to 8% (Table 2). Most of these deaths were ascribed to cardiovascular causes or infection; few were ascribed to cancer. Within 12 weeks, 19–29% of patients had been re-admitted, mostly for cardiovascular problems of which HF was the main reason in about half of admissions except for the group with an uncertain contribution of HF to the index admission.

**Fig. 1:**
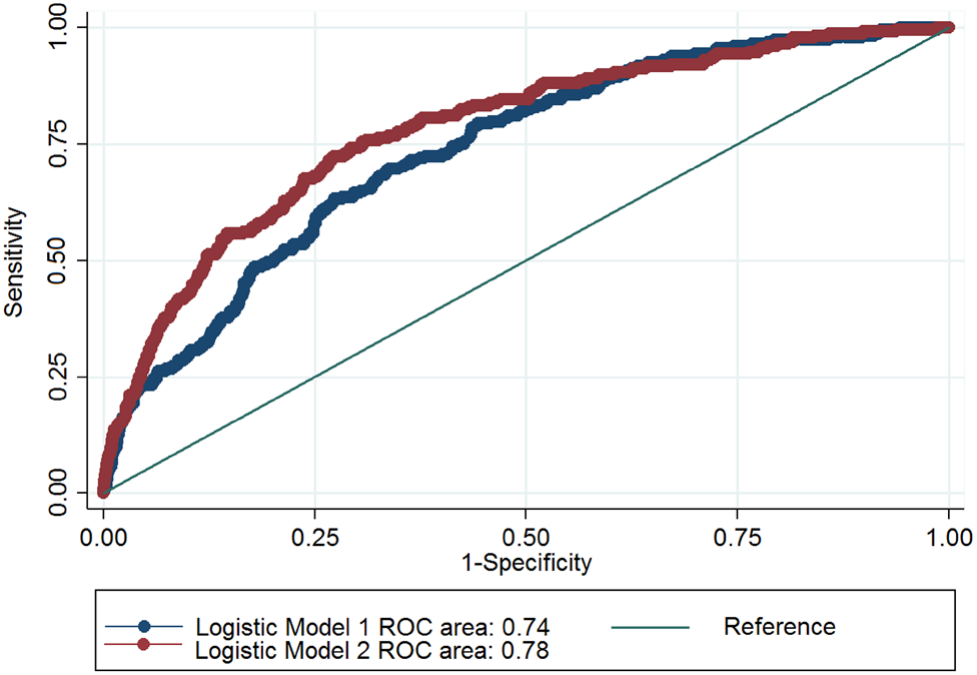
Comparison of two logistic regression models to assess mortality during index admission by ROC curves. *ROC* receiver operator characteristic

On multivariable analysis, MI during the index admission, a history of VT/VF, a history of stroke, left ventricular dilatation, and serum creatinine concentration were associated with mortality on the index admission (Table 3). The area under receiver operating characteristics (ROC) curve was 0.74. A second model, using a less rigorous *p* value of 0.1 for selection, identified four more variables; age, sex, medical history of hypertension and infection. The area under ROC curve in the second model was 0.78 (Fig. 1). We used Akaike’s information criterion (AIC) and Bayesian information criterion (BIC) to compare the two models. A smaller AIC/BIC ratio in model 2 (0.96 in model 1 and 0.95 in model 2) and a large difference of BIC between models (67) indicated the superiority of model 2 [19, 20]. In a final logistic regression model after adjusting for all relevant covariates, mortality remained higher in groups 1–6 as compared to group 7 (uncertain contribution of HF to admission).

**Table 3:** Logistic regression model for mortality during index admission

	Model 1 AUC 0.74			Model 2 AUC 0.78		
	Odds ratio as compare to class 7	*p* value	95% Confidence interval	Odds ratio as compare to class 7	*p* value	95% Confidence interval
Group 1	4.29	< 0.001	2.38–7.96	4.18	< 0.001	2.2–8.1
Group 2	3.58	< 0.001	1.97–6.51	4.08	< 0.001	2.14–7.79
Group 3	4.34	< 0.001	2.60–7.25	4.09	< 0.001	2.39–7.05
Group 4	3.54	< 0.001	2.33–5.39	3.27	< 0.001	2.10–5.09
Group 5	2.93	< 0.001	1.73–4.96	3.39	< 0.001	1.94–5.95
Group 6	2.24	0.009	1.23–4.08	2.90	0.001	1.54–5.48
Age^a^	–	–	–	1.03	< 0.001	1.01–1.04
Sex^a^	–	–	–	0.86	0.36	0.64–1.17
MI this admission	2.61	< 0.001	1.58–4.31	2.52	< 0.001	1.47–4.35
VT/VF (anytime)	2.85	< 0.001	2.02–4.02	3.10	< 0.001	2.15–4.49
H/O hypertension^a^	–	–	–	0.93	0.62	0.70–1.24
Stroke (anytime)	2.08	< 0.001	1.39–3.13	1.62	0.03	1.04–2.52
H/O infection^a^ (during this admission)	–			3.37	< 0.001	2.52–4.50
LV dilatation	0.95	0.73	0.71–1.27			
Creatinine	1.003	0.012	1.002–1.004	1.01	0.04	1.001–1.02

*AUC* area under curve, *MI* myocardial infarction, *VT* ventricle tachycardia, *VF* ventricle fibrillation, *H/O* history of, *LV* left ventricle</bib>

^a^Variables only included in model 2

For mortality between discharge and 12 weeks, a history of valve repair was the only variable which was significant using *p* < 0.05 for model selection in 25 cross validations and the model discrimination was poor (ROC 0.55). If *p* < 0.1 was used, a history of diabetes, LVSD, left ventricle dilatation, and history of angina during index admission provided additional information but this improved the ROC only to 0.57. In the logistic regression model, only Group 2 (ACS) (OR 1.73, *p* = 0.03, CI 1.07–2.95) and Group 4 (severe breathlessness) (OR 1.61, *p* = 0.002, CI 1.18–2.18) added to model prediction (OR compared to Group 7).

In a multivariable analysis investigating variables associated with all-cause readmission during the 12-week follow-up period, a history of hypertension, LVSD, and aortic stenosis were identified in 25 cross validations using *p* < 0.05 for model selection. In the logistic regression, only Group 6 added to the model with OR of 1.69 (*p* < 0.001, CI 1.34–2.12) compared to Group 7. However, the area under the ROC curve was only 0.55. When *p* < 0.1 was used to select variables from 25 cross validations, a history of infection, a history of valve replacement, and mitral regurgitation were identified that improved the ROC to 0.57 and again, only Group 6 added to the logistic regression model (OR of 1.77, *p* < 0.001, CI 1.40–2.24).

## Discussion

EHFS-1 was designed to investigate the overall burden of heart failure from a hospital perspective and not just a narrowly defined group of patients admitted with heart failure as a primary diagnosis and managed by cardiologists [3, 21]. The survey emphasizes the heterogeneity of patients hospitalized with suspected HF [22, 23]. Fewer than half of patients presented with severe breathlessness at rest and yet this has been the main focus of trials of AHF until now. Patients presenting primarily with increasing oedema appear to be a common, but neglected group of patients [24]; recent post-hoc analyses suggests that such patients might account for the response observed to agents for AHF such as serelaxin [25].

It is now fashionable to highlight the lack of progress in the treatment of AHF, as opposed to the huge progress made in the last 25 years for chronic HF [2, 26–28]. Unfortunately, the only Class 1, level of evidence A recommendation for the management of AHF in the ESC 2012 guidelines is thrombo-embolism prophylaxis, but it is reduced to Class 1, level of evidence B in ESC 2016 acute and chronic HF guidelines [29, 30]. In the most recent ESC guidelines (2016), no treatment was given a Class 1, level of evidence A recommendation for the management of AHF. There are many possible reasons for lack of progress in AHF [31]. The interventions studied may be truly ineffective or study design may have been inadequate [32, 33]. However, the heterogeneity of the patient population probably plays a major role. More precise patient selection, timing of intervention and targeting of therapy in clinical trials could reap large dividends [34]. More precise targeting does not necessarily mean more restrictive inclusion criteria. For instance, if congestion and peripheral oedema, which usually develops over several weeks, is the primary treatment target then there is little point in trying to enrol patients within a few hours of admission, which is logistically difficult from a research perspective and greatly reduces recruitment. Peripheral oedema often persists for many days after initiating treatment, allowing time for a new intervention to be introduced and its effects to become apparent. However, for patients with severe breathlessness due to pulmonary oedema, the onset is often abrupt; delaying intervention even for a few minutes may not be acceptable, and symptoms may have largely resolved within a few hours [2, 35]. There is only a small window of opportunity to show that a new intervention accelerates the improvement in symptoms. Whether health services would pay for a treatment that shortens the time to symptom relief by a few minutes is uncertain. Short-term treatments have not yet been shown to reduce the risk of longer term relapse or death.

Historically, the spectacular success in managing acute myocardial infarction (MI) was made possible by the development of coronary care units and the segregation of patients into ST elevation MI (STEMI) and non-ST elevation MI (NSTEMI) [36–38]. The same may be true for AHF not only in terms of their presentation, but also their underlying left ventricular phenotype and atrial rhythm and where they are managed [30, 39]. Most trials of AHF have enrolled a mixture of patients presenting with severe acute-onset breathlessness at rest (pulmonary oedema) and others with sub-acute worsening of peripheral congestion who are not breathless sitting upright at rest, but have orthopnoea and are breathless on minor exertion. The median time to enrolment in trials of AHF, with one exception, has never been less than 6 h, by which time most patients with acute pulmonary oedema have responded to a combination of diuretics and oxygen and have only residual symptoms [2, 40]. The one exception is the 3CPO study that enrolled patients with acute pulmonary oedema with heart and respiratory rates of 114 and 33, respectively, within a few hours of presentation [41].

Not unsurprisingly, patients presenting with cardiogenic shock, VT/VF, and ACS had a much worse in-hospital prognosis, but subsequent to discharge their prognosis, both in terms of readmissions and death, was rather similar to patients with other presentations. Clearly, these patients require urgent measures to correct the haemodynamic disturbance and to limit myocardial damage at the time of presentation. The short-term prognosis of patients with AHF reported in epidemiological studies will depend greatly on whether such patients are included. HF as a secondary, rather than primary, diagnosis may have a much worse short-term outcome, but few trials target such patients [18]. These findings highlight that HF is not a distinct diagnosis, but rather, a collection of clinical conditions with a great variety of underlying causes and clinical features, which fall under one umbrella term that is generally associated with a poor outcome. Targeting the therapeutic approach more accurately at specific patient subsets, presentations, aetiologies, and pathophysiologies and precipitating factors may yield greater benefit than a more generalised approach as is usual in current clinical trials. However, post-discharge outcome was rather similar regardless of presentation, even when it was unclear whether HF had precipitated or complicated the admission. Clinical trials often exclude patients who do not fulfil robust criteria for a diagnosis of HF but who, nonetheless, almost certainly have this diagnosis and also have a poor prognosis for whom either further investigation and better treatment is required [42].

Patient heterogeneity amongst patients presenting with AHF is a fundamental problem when designing and performing clinical trials. More accurate patient characterization for right treatment on right target is most crucial for success in clinical step for designs of new clinical trials and clinical practice. Clinical presentation, precipitating factors, underlying cardiac phenotype and aetiology of cardiac dysfunction might each be used to classify patients with the AHF and select them for trials. These episodes are main areas to be considered for better categorization of these patients. Preferably, these areas should be clearly identified before randomization to a new therapy. For example, patients presenting with pulmonary oedema due to ACS are likely to benefit from different treatments from those presenting with severe peripheral oedema and atrial fibrillation. Accurate classification requires a basic set of information including a medical history and physical examination, haematology and biochemistry profile, oxygen saturation, chest X-ray, an electrocardiogram, and an echocardiogram. However, urgent access to echocardiography is often problematic due to a lack of skilled personnel available either at unsociable hours or available on medical wards within a few hours or sometimes even days, if at all. Up skilling the medical workforce to provide at least basic echocardiography quickly and routinely should improve the management.

## Limitations

EHFS-1 was conducted in 2000-01, but, until now, there have been no innovations in therapy for AHF and recent trials suggest that the 12 week mortality of AHF has changed little in the past 15 years [16, 43, 44]. We developed mutually exclusive categories of patients but of course; in reality, some patients will belong to more than one class. However, in most such clinical situations, one presentation dominates. Attempting to avoid falling into the trap of capturing data on only narrowly defined ‘cardiological’ heart failure may have caused some confusion and inconsistent answers for our international group of investigators, especially for patients who were taking loop diuretics, but who had not been diagnosed with heart failure. However, it is impossible to assess the quality of care with respect to investigation if only patients who already have a definitive diagnosis of heart failure are included. Many patients hospitalized with a diagnosis or features suspicious of HF were admitted primarily for another reason. Most had features to suggest that they did indeed have HF and these patients had a high morbidity and mortality subsequent to discharge. This group of patients has lower in-patient mortality but only slightly lower rates of readmission and death subsequent to discharge compared to other groups, although their rates of readmission for worsening heart failure and of cardiovascular deaths were substantially lower.

A major limitation of this survey was the failure to specifically ask about peripheral oedema. We assume that when peripheral oedema was the major presentation, these will have been classified as ‘other’. However, many patients presenting with oedema will have had breathlessness on mild exertion or even at rest and may have been classified as presenting with breathlessness.

## Conclusion

Acute heart failure is a collection of syndromes with different clinical presentations, precipitating factors, underlying aetiology, and pathophysiology that may affect the clinical course, prognosis, and response to treatment. A common feature of AHF is poor outcomes and the unmet clinical need is still great. Focussing on specific presentations, such as acute pulmonary oedema, worsening peripheral oedema, or rapid AF, that may differ in their pathophysiology and treatment goals might succeed, where the current efforts have so far failed.
